# Prehospital intravenous access and fluid resuscitation in severe sepsis: an observational cohort study

**DOI:** 10.1186/s13054-014-0533-x

**Published:** 2014-09-27

**Authors:** Christopher W Seymour, Colin R Cooke, Susan R Heckbert, John A Spertus, Clifton W Callaway, Christian Martin-Gill, Donald M Yealy, Thomas D Rea, Derek C Angus

**Affiliations:** Departments of Critical Care Medicine and Emergency Medicine, University of Pittsburgh School of Medicine, 3550 Terrace Street, 15261 Pittsburgh, PA USA; Clinical Research, Investigation, and Systems Modeling of Acute Illness (CRISMA) Center, 3550 Terrace Street, 15261 Pittsburgh, PA USA; Division of Pulmonary and Critical Care Medicine, University of Michigan, Ann Arbor, MI USA; Center for Healthcare Outcomes & Policy, University of Michigan, Ann Arbor, MI USA; Department of Epidemiology, University of Washington School of Public Health, Seattle, WA USA; Saint Luke’s Mid America Heart Institute, University of Missouri-Kansas City School of Medicine, Kansas City, USA; Department of Emergency Medicine, University of Pittsburgh School of Medicine, Pittsburgh, PA USA; King County MedicOne, King County Emergency Medical Services, Seattle, WA USA; Department of Medicine, Division of General Internal Medicine, University of Washington School of Medicine, Seattle, WA USA; Department of Critical Care Medicine, University of Pittsburgh School of Medicine, Pittsburgh, PA USA

## Abstract

**Introduction:**

Prompt treatment of severe sepsis in the Emergency Department reduces deaths, but the role of prehospital fluid resuscitation is unknown. We sought to determine the risk-adjusted association between prehospital fluid administration and hospital mortality among emergency medical services (EMS) patients admitted with severe sepsis.

**Methods:**

We performed a prospective, observational study of patients hospitalized with severe sepsis on admission among 45,394 adult EMS encounters taken to 15 hospitals from 11/2009 to 12/2010 by a two-tier EMS system in King County, Washington. The region mandated recording of prehospital intravenous catheter and fluid administration in prehospital records, along with detailed demographic, incident, physiologic, and hospital adjustment variables. We determined the effect of prehospital intravenous catheter or fluid versus no catheter or fluid on all-cause mortality using multivariable logistic regression.

**Results:**

Of all encounters, 1,350 met criteria for severe sepsis on admission, of whom 205 (15%) died by hospital discharge, 312 (23%) received prehospital intravenous fluid, 90 (7%) received a prehospital catheter alone and 948 (70%) did not receive catheter or fluid. EMS administered a median prehospital fluid volume of 500 mL (interquartile range (IQR): 200, 1000 mL). In adjusted models, the administration of any prehospital fluid was associated with reduced hospital mortality (Odds ratio =0.46; 95% Confidence interval: 0.23, 0.88; *P* =0.02) compared to no prehospital fluid. The odds of hospital mortality were also lower among severe sepsis patients treated with prehospital intravenous catheter alone (Odds ratio =0.3; 95% Confidence interval: 0.17 to 0.57; *P* <0.01).

**Conclusions:**

In a population-based study, the administration of prehospital fluid and placement of intravenous access were associated with decreased odds of hospital mortality compared with no prehospital catheter or fluid.

**Electronic supplementary material:**

The online version of this article (doi:10.1186/s13054-014-0533-x) contains supplementary material, which is available to authorized users.

## Introduction

Prompt recognition and treatment of severe sepsis and septic shock are keystones of optimal care [1–4]. Such early therapy reduces both absolute and relative hypovolemia and subsequent organ dysfunction. The current focus of sepsis resuscitation trials is in the emergency department [5], yet many septic patients receive prehospital care from emergency medical services (EMS) [6]. Although sepsis is commonly encountered by EMS [6], the administration of prehospital intravenous fluid is variable [7,8]. Prehospital fluid could alter sepsis outcomes by directly improving organ perfusion or by altering the process of care after arrival at the emergency department (ED) [9]. The latter is noted in the care of ST elevation myocardial infarction, where EMS electrocardiogram acquisition is a key factor in improving the response after ED arrival [10]. While prehospital resuscitation has been rigorously tested in other time-sensitive syndromes, such as trauma and cardiac arrest [11–13], no trial has explored the benefit or harm of fluid resuscitation in prehospital sepsis.

We sought to examine if prehospital fluid administration is associated with hospital mortality in adults with severe sepsis. We examined if either prehospital intravenous catheter or fluid is associated with reduced mortality compared to no intravenous catheter or fluid and tested these associations among septic patients with prehospital hypotension.

## Materials and methods

### Study design and participants

We examined prospectively collected, population-based data from King County, WA, USA, EMS [14]. We studied all eligible EMS patients 18 years old or older transported to an acute care hospital in King County who were admitted with severe sepsis diagnosed as ‘present on admission’ from 1 November 2009 to 31 December 2010. Eligible EMS patients were those within the catchment of King County Medic One – one of the five advanced life support EMS agencies in King County, WA. We excluded prehospital subjects with trauma or those suffering cardiac arrest. We defined severe sepsis cases in the administrative hospital record used the Angus International Classification of Diseases, Ninth Revision, Clinical Modification (ICD9-CM) implementation (infection + organ dysfunction) as it is the most accurate and sensitive algorithm compared to structured manual chart review [15]. We also included any subjects with explicit diagnoses of severe sepsis (ICD-9-CM 995.92 and 785.52). We defined severe sepsis diagnosed on admission if both the ICD-9-CM code for infection and organ dysfunction, or explicit severe sepsis code had ‘present on admission’ indicator flags. We previously validated this definition using manual review [6]. Repeat EMS encounters for individual patients were included in the cohort. The study was approved by the Institutional Review Boards for the Washington State Department of Health, King County Emergency Medical Services, and the University of Washington with waiver of informed consent and HIPAA authorization.

### Study setting

King County Medic One serves a population of approximately 750,000 persons who reside in urban, suburban and rural areas over approximately 600 square miles. The EMS system responds to medical emergencies in two tiers. The first tier is comprised of 14 EMS agencies, which comprise firefighter emergency medical technicians trained in basic life support (BLS). The second tier is comprised of a single paramedic agency, which is trained in advanced life support including electrocardiogram rhythm interpretation, intravenous and intraosseous line placement, medication administration and endotracheal intubation. The second tier is dispatched to patients with more severe illness according to criteria-based dispatch assessment or first-tier EMS patient evaluation. Patients are transported to one of 15 hospitals.

The triage and care of potential sepsis patients by EMS is at the discretion of the responding personnel. There is no formal sepsis protocol; rather the paramedic discusses cases with an online medical control emergency physician to determine treatment (including intravenous fluids) and disposition. Paramedics are required to perform 35 intravenous catheter starts annually to remain eligible for certification. Only lactated ringers was available for intravenous (IV) fluid during the study period and BLS providers cannot start IV catheters or give IV fluid. No antibiotics were administered by EMS.

### Data sources and quality control

The King County EMS electronic database is drawn from written and electronic medical incident report forms completed by EMS personnel. Prior to study start, we prospectively modified the medical incident report form such that any paramedic documenting an IV catheter start during the study period is also required to record the administration of prehospital fluid (yes/no) and total prehospital volume (mL). The database was auto-populated with prehospital time intervals and geospatial data from the dispatch center. We linked EMS records to the Washington State Comprehensive Hospital Abstract Reporting System (CHARS) database from 2009 to 2011. CHARS is a state-wide database of all hospitalizations, with accurate diagnostic, procedural and discharge data, including present-on-admission indicators [16]. Prehospital and CHARS data were linked using a manually validated, hierarchical matching algorithm using direct identifiers with more than 86% match success [14].

### Study exposures and confounders

We defined prehospital resuscitation in three categories: 1) no IV catheter placed and no fluid administered; 2) IV catheter placed but no fluid administered; and 3) IV catheter placed and fluid administered. We evaluated the placement of IV catheter as a separate category as prior data suggest the procedure may impact patient outcomes in the prehospital critically ill [17], perhaps through modifying care processes in the ED [18]. We included the placement of any type of prehospital IV catheter, peripheral, central or intraosseous, as documented by EMS in the medical incident report form. Data were unavailable on catheter starts that were unsuccessful, or other intravenous medications co-administered. We abstracted multiple potential confounders based on clinical relevance and prior literature [14,17]. These included age (years), sex, and initial vital signs as documented by first arriving EMS personnel - respiratory rate (RR, breaths/minute), heart rate (HR, beats/minute), systolic blood pressure (SBP, mmHg), pulse oximetry (SaO_2_,%), and Glasgow Coma Scale score (GCS) when available. We abstracted an index of illness severity as determined by EMS personnel: ‘life-threatening’ , ‘urgent’ , and ‘non-urgent’. We defined prehospital location as home, nursing home or medical facility, and abstracted additional prehospital procedures, such as electrocardiogram monitoring, delivery of supplemental oxygen, bag-valve mask ventilation, or intubation. We defined primary prehospital diagnostic categories as respiratory, cardiovascular or neurologic, and calculated a validated prehospital critical illness severity score [14,19]. We defined mode of transport from scene to hospital as advanced life support (ALS) or BLS, and we abstracted standard prehospital time intervals (minutes) [20], including call receipt to unit notification, notification to unit responding, unit responding to arrival at scene, total scene time and scene to hospital arrival.

### Primary outcomes

The primary endpoint was hospital mortality, defined by disposition in the hospital discharge data. We studied additional *a priori* secondary outcomes including ICU admission and increasing organ failures during hospitalization (for example, number of total hospital organ failures at discharge minus number of organ failures defined as present on admission). We used only organ failures as defined by the Angus sepsis implementation [15].

### Missing data

Missing data in the King County MedicOne database was variable, for example, ranging from 0% for age to 45% for pulse oximetry [14,17]. We assumed missing data were conditional on observed covariates, and we performed multiple imputation for all missing values using a regression switching approach (multiple imputation by chained equations) [21]. More detail is provided in Additional file 1: Methods.

### Statistical analyses

We compared prehospital characteristics and outcomes of subjects with descriptive characteristics across three treatment groups: 1) no IV catheter placed and no fluid administered; 2) IV catheter placed but no fluid administered; and 3) IV catheter placed and fluid administered. We report continuous variables as mean (standard deviation (SD)) or median (interquartile range (IQR)), as appropriate; categorical variables are reported as frequencies or percentages. To illustrate current practice, we plotted the predicted volume of intravenous fluid administration (with 95% confidence interval) across initial prehospital SBP using crude linear regression models.

Our analysis had four steps: 1) primary regression modelling of prehospital catheter and fluid on mortality; 2) *a priori* sensitivity analyses in restricted groups’ 3) simulation of unmeasured confounders; and 4) falsification analyses to test for spurious results.

In the primary analysis to determine if prehospital catheter and fluid was associated with a lower risk of mortality (yes/no), we built a series of logistic regression models with robust (Huber-White) standard errors for regression coefficients, including unadjusted, adjusted for selected variables and adjusted for all covariates. We used *a priori*-determined confounders guided by past literature and theory [14,17], including demographics (age, sex), prehospital location (nursing home, medical facility, home), initial vital signs (HR, RR, SaO_2_, GCS, SBP), EMS diagnostic category, EMS severity index, mode of transport to receiving hospital, prehospital time intervals (responding to scene, total scene, leave scene to hospital) and additional EMS procedures (electrocardiographic monitoring, bag-valve mask ventilation, intubation, supplemental oxygen). We used a broad spectrum of measured confounders, including clustering variables, to account for treatment selection (for example, when paramedics might select prehospital fluid for patients based upon a confounding variable, such as illness severity). We used independent imputed datasets for all models and combined estimates using Rubin’s rules [22]. We used generalized estimating equations to account for the non-independence of mortality across hospitals [23]. We then tested for the association between prehospital fluid and/or catheter and our secondary outcomes – increasing organ failures during hospitalization (yes/no) and ICU admission at any time during hospitalization (yes/no). We used the *margins* command to determine the predictive margins for each treatment group at observed covariate values [24].

### Sensitivity analyses and unmeasured confounding

We performed several sensitivity analyses to test the robustness of our results. First, we restricted to patients in whom ALS participated in care. This analysis limited confounding by severity [25]. Second, we repeated the analysis only among those with prehospital hypotension (initial SBP less than 110 mmHg) [26], as these patients may be most likely to benefit from prehospital resuscitation and have greater mortality. Finally, we determined the magnitude of a hypothetical, unmeasured confounder needed to account for the benefit of prehospital fluid using quantitative bias analysis [27]. We varied the effect of this hypothetical confounding variable on hospital mortality and its prevalence among no catheter/no fluid patients, in order to determine how the association between fluid resuscitation and mortality would change after adjustment. In these analyses, we assumed: 1) the prevalence of the unmeasured confounder among patients receiving prehospital intravenous access/fluid was 5.0%; 2) no modification of the effect of prehospital fluid by the unmeasured confounder; and 3) the confounder was uncorrelated with other variables in the model. This approach reveals how strong and common the unmeasured confounder must be to abrogate the potential treatment effect of prehospital catheter and fluid on hospital mortality.

### Falsification analyses

We used pre-specified falsification analyses to strengthen the validity of observational research and uncover spurious associations [28]. Our approach used two prevalent, nonsensical outcomes: 1) admission month is prime number; and 2) sum of age integers is even (that is, 24 year old =6 = ‘even’). We then re-ran our identical primary model on these two outcomes. Then, we replaced our prehospital fluid exposure with nonsensical exposures (for example, ‘admission month is prime number’ and ‘age is even’) and repeated our identical primary model with the outcome of hospital mortality. These models can help support the validity of the primary models, but are not intended to imply causality (14).

All analyses were performed with STATA 11.0 (StataCorp, College Station, TX, USA). All tests of significance used a two-sided *P* ≤0.05.

## Results

### Baseline characteristics

**Figure 1:**
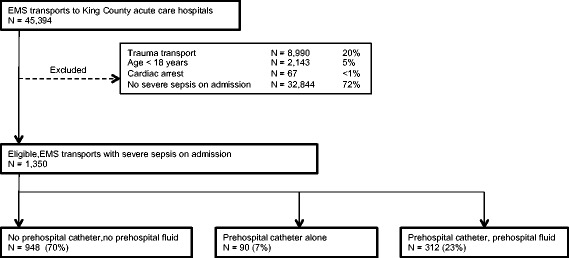
**Subject accrual.**

A total of 45,394 eligible EMS encounters were transported to a hospital during the study period (Figure 1), of whom 1,350 were diagnosed with severe sepsis as present on admission. Less than one third received prehospital IV catheter and fluid (N =312, 23%), and fewer had a catheter alone (N =90, 7%). Most had no catheter placed or fluid administered (N =948, 70%). Severe sepsis patients receiving fluids were similar in age, sex and prehospital location compared to other groups (Table 1). However, severe sepsis patients administered prehospital fluids more frequently had prehospital hypotension, a lower GCS score and an assessment of ‘life-threatening conditions’ by EMS. Subjects who received fluid were administered a median 500 mL (IQR: 200, 1000 mL) and greater volumes for more severe prehospital hypotension (Figure S1, Additional file 1). Subjects receiving an IV catheter alone more often had a paramedic diagnosis of a respiratory condition and prehospital tachypnea. Overall, sicker subjects received prehospital catheter or fluids, as measured by mean prehospital critical illness scores, the proportion transported by ALS, and longer scene time (Table 1).

**Table 1:** **Characteristics and outcomes of severe sepsis patients (N =1,350) across three groups**

**Variable**	**No catheter or fluid**	**Catheter alone**	**Catheter and fluid**
Number (%)	948 (70)	90 (7)	312 (23)
Age, mean (SD) years	71 (15)	73 (13)	68 (16)
Male, number (%)	409 (43)	45 (51)	145 (47)
Prehospital location, number (%)			
Nursing home	65 (7)	4 (4)	17 (5)
Medical facility	143 (15)	16 (18)	59 (19)
Home	676 (71)	67 (74)	212 (68)
Paramedic diagnostic category, number (%)			
Cardiovascular	53 (5)	23 (26)	65 (21)
Respiratory	194 (20)	46 (51)	104 (33)
Neurological	131 (14)	6 (7)	29 (9)
EMS severity index, number (%)^a^			
Life-threatening	3 (2)	24 (27)	108 (35)
Urgent	49 (33)	57 (64)	173 (56)
Non-urgent	95 (64)	8 (9)	30 (10)
Prehospital critical illness score, mean (SD)	1.72 (0.93)	3.1 (0.89)	3.2 (0.90)
Prehospital intervals, mean (SD) minutes			
Response to scene	6.3 (3.2)	7.0 (4.3)	6.3 (3.4)
Arrived at scene to cleared scene	25.8 (11.3)	42.9 (18.6)	45.4 (18.8)
Cleared scene to hospital	14.0 (10.2)	9.2 (4.2)	10.6 (6.6)
Prehospital vital signs			
Systolic blood pressure ≤110 mmHg, number (%)	288 (30)	15 (16)	155 (50)
Systolic blood pressure, mmHg mean (SD)	124 (34)	147 (43)	117 (48)
Respiratory rate, breaths per min, mean (SD)	21 (7)	29 (11)	25 (12)
Heart rate, beats per minute, mean (SD)	93 (22)	103 (30)	103 (31)
Glasgow Coma Scale score, mean (SD)	13.6 (2.8)	12.4 (4.2)	11.1 (4.5)
Prehospital interventions, number (%)			
Intubation	1 (<1)	34 (38)	117 (38)
Bag valve mask ventilation	4 (<1)	33 (37)	104 (33)
EKG monitoring	147 (16)	88 (98)	307 (98)
Supplemental oxygen	394 (42)	90 (100)	311 (100)
Transport from scene, number (%)			
Advanced life support	30 (3)	83 (92)	262 (86)
Basic life support	80 (9)	0 (0)	4 (1)
Private ambulance	799 (88)	7 (8)	39 (13)
Outcomes, number (%)			
Organ failures present, mean (SD)	1.4 (0.9)	1.8 (1.2)	2.1 (1.4)
ICU admission	405 (43)	64 (71)	243 (78)
Hospital mortality	120 (13)	14 (16)	71 (23)

^a^EMS Severity index determined by first arriving EMS clinician, as part of routine assessment with vital signs. EKG, electrocardiogram; EMS, emergency medical services; ICU, intensive care unit; IQR, interquartile range; SD, standard deviation.

### Primary analysis

**Figure 2:**
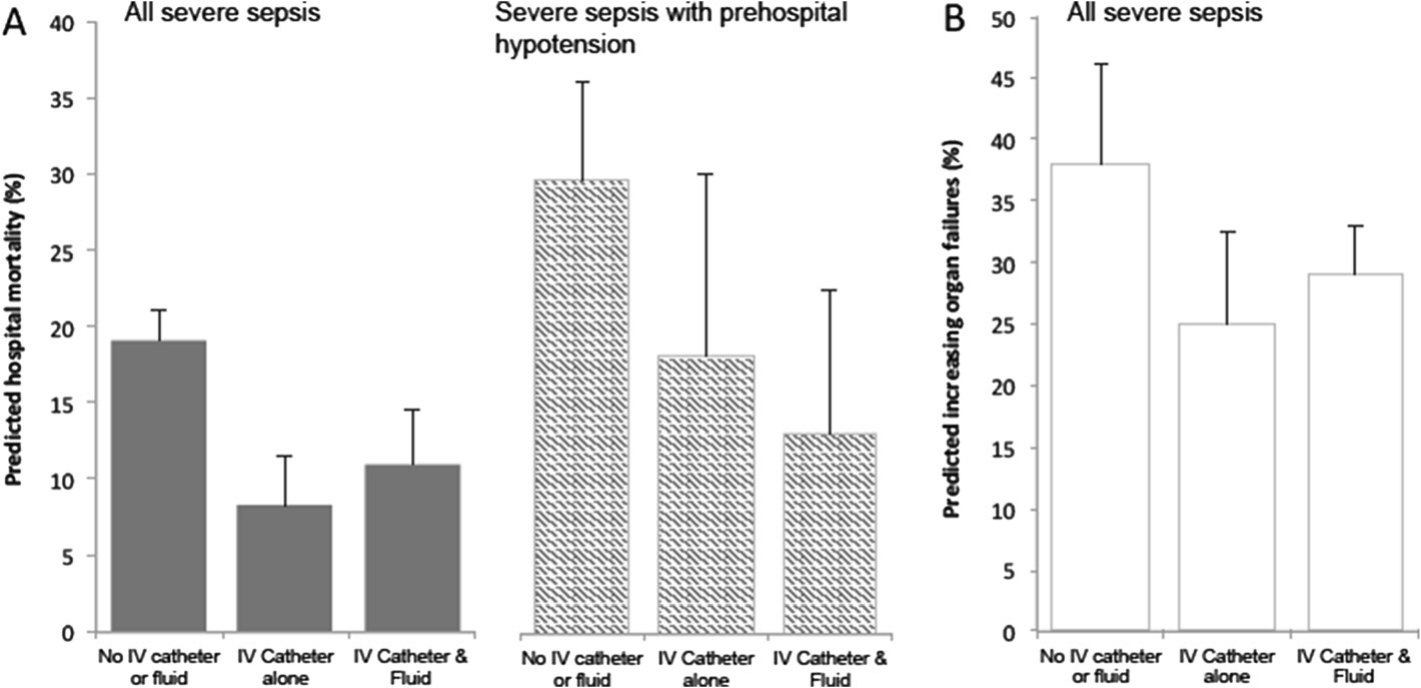
**Predicted hospital mortality from fully adjusted models. (A)** Predicted hospital mortality from fully adjusted models for subjects with severe sepsis (grey bars*,* N =1,350) and those with prehospital hypotension (<=110 mmHg, hashed bars*,* N =554), **(B)** predicted rate of increasing organ failures during hospitalization (empty bars*,* N =1,350). Bars represent estimates at observed covariates values for each exposure group: no intravenous access or catheter versus intravenous catheter alone versus intravenous catheter and fluid; error bars represent 95% confidence intervals.

In unadjusted regression models (Table 2), we found that both prehospital catheter alone (OR =1.27, 95% CI: 0.71, 2.28) and prehospital catheter and fluid (OR =2.05, 95%CI: 1.72, 2.46) were associated with hospital mortality. Partial adjustment for demographics and selected physiologic variables attenuated this association (Table 2), and full adjustment uncovered that prehospital catheter and fluid (OR =0.46, 95% CI: 0.23, 0.88) and prehospital catheter alone (OR =0.31, 95% CI: 0.17, 0.57) were associated with a reduction in the odds of hospital mortality compared to no prehospital fluid and no catheter. After full adjustment, we also observed that prehospital catheter and fluid was associated with reduced odds of increasing organ failures during hospitalization (OR =0.58, 95% CI: 0.34, 0.98). The association of prehospital fluid and catheter with ICU admission did not reach statistical significance (OR =0.64, 95% CI: 0.37, 1.10). The predicted risk of hospital mortality and increasing organ failures from adjusted models are shown in Figure 2.

**Table 2:** **Odds ratio (95% CI) for hospital mortality across treatment groups derived from unadjusted, partial and fully adjusted logistic regression models**

**Number (%) with outcome**						
**Model**	**All patients (Number = 1350)**	**No catheter or fluid (Number = 948)**	**Catheter only (Number = 90)**	**Catheter and fluid (Number = 312)**	**Odds ratio (95% CI) for catheter alone** ^**a**^	**Odds ratio (95% CI) for catheter and fluid** ^**a**^
Hospital mortality number (%)						
Unadjusted	205 (15)	120 (13)	14 (16)	71 (23)	1.27 (0.71, 2.28)	2.05 (1.72, 2.46)
Adjusted for select variables^b^					0.98 (0.51, 1.86)	1.26 (0.98, 1.63)
Adjusted for all covariates^c^					0.31 (0.17, 0.57)	0.46 (0.23, 0.88)
Increasing organ failures during hospitalization						
Unadjusted	485 (36)	265 (28)	47 (52)	173 (55)	2.72 (1.90, 3.90)	3.05 (2.67, 3.49)
Adjusted for select variables^b^					1.34 (0.49, 3.67)	1.66 (0.87, 3.17)
Adjusted for all covariates^c^					0.43 (0.21, 0.90)	0.58 (0.34, 0.98)
ICU admission						
Unadjusted	712 (53)	405 (42)	64 (71)	243 (78)	3.35 (2.03, 5.58)	4.50 (3.63, 5.59)
Adjusted for select variables^b^					1.81 (1.03, 3.17)	4.50 (3.63, 5.59)
Adjusted for all covariates^c^					0.41 (0.24, 0.70)	0.64 (0.37, 1.10)

^a^Compared to referent group: no intravenous catheter or fluid; ^b^partial adjustment variables include age, gender, and initial prehospital heart rate, respiratory rate, Glasgow Coma Scale score, pulse oximetry, systolic blood pleasure; ^c^full adjustment includes partial adjustment variables, transport mode from scene, total scene time, transport time to hospital, prehospital procedures (for example, intubation, EKG monitoring, supplemental oxygen, bag-valve mask ventilation), EMS disease category (for example, cardiac, neurologic, respiratory), EMS call urgency (for example, life threatening, urgent, non-urgent). All estimates used imputed data after Rubin’s rules, including generalized estimating equations to account for within hospital clustering. Models shown for primary and secondary outcomes. CI, confidence interval; EKG, electrocardiogram; EMS, emergency medical services.

### Sensitivity analyses

Among subjects with prehospital hypotension (N =554), prehospital fluid and catheter (OR =0.26, 95% CI: 0.08, 0.86) was also associated with lower odds of hospital mortality (Figure 2). When restricting to subjects cared for by ALS (N =549), prehospital fluid and catheter (OR =0.31, 95% CI: 0.15, 0.66) and catheter alone (OR =0.24, 95% CI: 0.14, 0.39) similarly reduced the odds of hospital mortality. In quantitative bias analysis (Table 3), we observed that a hypothetical confounder must be at least four times as prevalent among those without catheter or fluid (compared to those who received both), and the odds of hospital mortality among subjects with the confounder must be more than 2.0 for the adjusted risk of mortality to appear equivalent between patients who received prehospital fluid versus no catheter or fluid.

**Table 3:** **Quantitative bias analysis illustrating the odds of mortality under varying assumptions**

**Odds ratio of hypothetical confounder** ^**a**^	**Prevalence of unmeasured confounder among patients not receiving prehospital catheter or fluid**			
	**0.075**	**0.1**	**0.2**	**0.3**
1.25	0.46 (0.23,0.88)	0.46 (0.23, 0.89)	0.47 (0.24, 0.92)	0.48 (0.24, 0.94)
1.50	0.46 (0.23, 0.89)	0.46 (0.23, 0.90)	0.48 (0.24, 0.95)	0.50 (0.26, 0.99)
1.75	0.46 (0.23, 0.90)	0.47 (0.64, 0.92)	0.50 (0.25, 0.98)	**0.53 (0.27, 1.04)**
2.00	0.46 (0.23, 0.90)	0.47 (0.24, 0.93)	**0.51 (0.26, 1.01)**	**0.56 (0.28, 1.09)**
2.25	0.46 (0.24, 0.91)	0.48 (0.24, 0.94)	**0.53 (0.27, 1.04)**	**0.58 (0.30, 1.14)**
2.50	0.47 (0.24, 0.91)	0.48 (0.24, 0.95)	**0.54 (0.28, 1.07)**	**0.61 (0.31, 1.19)**

^a^Assumptions: 1) prevalence of unmeasured confounder among patients receiving prehospital fluid = 0.05; 2) no modification of the effect of prehospital fluid by the unmeasured confounder; 3) confounder uncorrelated with other variables in the model. The upper most left-hand corner is the observed odds ratio and bolded cells are conditions in which the observed odds ratio is no longer significant.

Finally, we performed multiple pre-specified falsification analyses (Table 4). These revealed that our model structure and exposures did not uncover erroneous associations with nonsensical outcomes such as ‘admission month is a prime number’ (OR =0.93, 95% CI: 0.60, 1.45). We similarly found no association in our models between nonsensical exposures and hospital mortality.

**Table 4:** **Falsification analyses with identical model structure and adjustment variables as primary model** ^**a**^

**Pre-specified falsification analyses**	**Odds ratio (95% CI) for catheter alone**	**Odds ratio (95% CI) for catheter and fluid**
**Nonsensical outcomes with identical exposures and model** ^***b***^		
Sum of age integers is an even number	0.75 (0.51, 1.10)	1.02 (0.82, 1.29)
Admission month is a prime number	1.11 (0.76, 1.60)	0.93 (0.60, 1.45)
**Nonsensical exposures with identical model and outcome** ^**a**^	**Odds ratio (95% CI)**	
Age is even number	0.92 (0.66, 1.27)	
Admission month is a prime number	0.90 (0.67, 1.20)	

^a^Identical models as above except intravenous catheter and fluid indicator variables removed and replaced with non-sensical exposures; ^b^all estimates use imputed data after Rubin’s rules, including generalized estimating equations to account for within hospital clustering. Referent category for odds ratios in nonsensical outcomes analysis was patients who received no catheter and no fluid. We tested both nonsensical outcomes and nonsensical exposures in separate analyses. CI, confidence interval.

## Discussion

In this population-based, observational cohort study of prehospital medical patients admitted with severe sepsis, we found that prehospital fluid administration is associated with a reduced odds of hospital mortality compared to no prehospital fluid, after multivariable adjustment. Similar to prior studies [17], the placement of a prehospital intravenous catheter alone was also associated with a reduction in the odds of hospital mortality. These results were robust to several sensitivity analyses that restricted the cohort, simulated the effect of various unmeasured confounders and tested *a priori* falsification endpoints.

Extending prior work in the ED [1,4], we found an association between prehospital fluid administration and patient outcomes in sepsis. This is consistent with prior hypothesis-generating data (without adjustment for treatment selection) that suggest prehospital fluid in sepsis may reduce time to goal mean arterial pressure after hospital arrival [18]. One mechanism for the treatment effect could be direct improvement in organ perfusion as average fluid volumes were 500 mL; our subgroup analysis among patients with prehospital hypotension supports this hypothesis, yet we could not assess relative changes in hemodynamic parameters after ED arrival. Notably, patients receiving prehospital fluids had reduced odds of developing new organ failures during hospitalization compared to those without prehospital fluid administration.

In addition, prehospital patients with severe sepsis who received intravenous access alone were also found to have lower odds of hospital mortality. This treatment effect may result from faster triage and initiation of care in the ED. Others report that arrival by EMS reduces time to antibiotics and fluids in the ED compared to sepsis patients who arrive by non-EMS [9]. A similar priming of the ED to deliver faster care is seen in EMS patients with suspected ST-elevation myocardial infarction or stroke [10,29]. It is also possible that the association of prehospital fluid or catheter with mortality could be an artefact of unmeasured factors, such as paramedic judgment and skill. Survey data suggest that paramedics’ knowledge and awareness of sepsis is widely variable [30].

These data have important methodological and research implications. First, large treatment effects uncovered in observational studies may not always be confirmed in subsequent randomized trials [31]. Despite multiple steps to account for biases in our design, a randomized experimental design would better elicit the true causal relationship between prehospital fluids, intravenous access and outcomes in sepsis. Such a step led to important findings about prehospital resuscitation in traumatic shock [13], but faces regulatory, ethical and logistical barriers. Our data are also not robust enough to understand how the dose of prehospital fluid changes outcomes, nor the choice of fluid or chloride content [32]. Finally, the ideal rate of infusion and potential for later complications including pulmonary edema or need for renal replacement therapy are key next targets for future study [11,32].

For clinicians, the findings do not mandate that EMS begin aggressively resuscitating septic patients. Rather, the data suggest that the prehospital phase of care may be the target of future intervention trials in sepsis; such trials would be improved if there were consistent operational criteria to identifying sepsis cases. Given the variable knowledge and awareness of sepsis among emergency care providers [30], clinicians should be urged to focus on sepsis education, developing protocolized alerts, decision aids, or coordinating recognition of sepsis across EMS, the ED, and the ICU. Because sepsis awareness is so poor among the general public [33], the burden is on frontline providers to suspect sepsis.

Out findings should be interpreted in the context of several potential limitations. First, our study did not randomly assign patients to specific prehospital resuscitation interventions. Thus, our results may be biased from a variety of measured and unmeasured confounders, such as the clinical acumen or skill of EMS with sepsis care. We determined through bias analysis that unmeasured confounders must be highly different between groups and strongly associated with hospital mortality for our findings to be rejected. We did not use propensity scores as they suffer the same limitations from unmeasured confounding, while instrumental variable analysis was considered but rejected due to the absence of a suitable instrument. We also acknowledge that different discharge practices across hospitals could bias the primary outcome of hospital mortality [34] and, thus, included hospital level clustering in our models. Fixed time point mortality at 90-days may be a more patient-centered outcome for future studies of prehospital resuscitation [35]. Because we identified severe sepsis cases on admission, we may have excluded some if prehospital fluid fully treated organ failure prior to ED arrival. Such misclassification would likely bias towards the null if these patients were included, as reduced organ failures are in the causal pathway of the proposed treatment benefit. We used administrative data for case finding, but chose the most valid ICD-9-CM algorithm for severe sepsis [15]. Although unsuccessful prehospital catheter placement is uncommon [36], we could not measure whether EMS intended to resuscitate but were unable. Our data also derive from a US EMS agency without specific sepsis protocols and instead uses online medical control, and so may not generalize to other EMS systems where physicians participate directly in care, transport times are different, or where prehospital sepsis protocols exist. Finally, data unavailable on the care delivered in the ED could help uncover potentially causal pathways for the associations we observed.

## Conclusions

In summary, we observed that prehospital fluid administration and intravenous catheter placement were associated with decreased odds of hospital mortality in medical patients with severe sepsis. Given the adverse consequences of time delays in sepsis, these data suggest that the prehospital phase of care may be an important opportunity for early resuscitation.

## Key messages

- Prehospital fluid administration and intravenous access were uncommon among EMS patients hospitalized with community severe sepsis
- Mean prehospital fluid administration in severe sepsis was 500 mL
- Placement of intravenous access and administration of fluid was associated with a significant reduction in the odds of hospital mortality compared to no fluid or intravenous access among severe sepsis patients
- The prehospital phase of care may be an important opportunity to test early fluid administration strategies in severe sepsis

## Additional file

Additional file 1:
**Methods.** Description of multiple imputation methods. **Figure S1.** Predicted volume of intravenous fluid across range of prehospital systolic blood pressure.

## Abbreviations

ALS: advanced life support
CHARS: comprehensive hospital abstract reporting system
CI: confidence interval
ED: emergency department
EMS: emergency medical services
GCS: Glasgow Coma Scale
HR: heart rate
ICU: intensive care unit
IQR: interquartile range
IV: intravenous
RR: respiratory rate
SBP: systolic blood pressure
SD: standard deviation
