# Supplementary material for: Prehospital intravenous access and fluid resuscitation in severe sepsis: an observational cohort study
Source: Crit Care. 2014 Sep 27;18(5):533. doi: 10.1186/s13054-014-0533-x (PMC4212132; doi:10.1186/s13054-014-0533-x)
Supplement: Additional file 1: — Methods. Description of multiple imputation methods. Figure S1. Predicted volume of intravenous fluid across range of prehospital systolic blood pressure. [file 13054_2014_533_MOESM1_ESM.docx]

**Additional File:**

**eMethods.** Description of multiple imputation methods

**eFigure 1.** Predicted volume of intravenous fluid across range of prehospital systolic blood pressure.

**References**

**eMethods. Multiple imputation methods**

**Multiple imputation procedure**

The multiple imputation with chained equations (MICE) approach generates values for all missing data conditional on the observed data for all patients. This assumes the missing data was “missing at random.” We used 10 cycles of regression switching to create each of 11 independent datasets. We included all model covariates and our primary outcome in the imputation procedure. For continuous, non-normal variables with upper and lower bounds, we used predictive mean matching (e.g. prehospital time intervals, Glasgow coma scale score, pulse oximetry).[^1^](#_ENREF_1) We modeled the EMS severity index, transport mode, and prehospital location(s) using multinominal logistic regression.]

**Analysis after multiple imputation**

We ran all our models on multiply imputed data (11 datasets). Regression coefficients and standard errors were combined using Rubin’s rules.[^2^](#_ENREF_2)

**eFigure 1**

**eFigure 1**. Predicted volume of prehospital intravenous fluid (mL) across range of initial prehospital systolic blood pressure among 312 severe sepsis subjects who received intravenous fluid. Estimates and 95% confidence intervals (grey) were derived from crude linear regression model.

**References**

**1.** Marshall A, Altman DG, Royston P, Holder RL. Comparison of techniques for handling missing covariate data within prognostic modelling studies: a simulation study. *BMC Med Res Methodol.* Jan 19;10(1):7.

**2.** Rubin DB. *Multiple imputation for nonresponse in surveys*. New York: John Wiley and sons; 2004.
